# Understanding the Use of Social and Emotional Learning in Elementary Schools: A Theory of Planned Behaviour Perspective

**DOI:** 10.3390/ejihpe15040048

**Published:** 2025-03-27

**Authors:** Mélanie Tinnes-Vigne, Claude Houssemand, Frédéric Guay, Débora Poncelet, Christophe Dierendonck

**Affiliations:** 1 Department of Education and Social Work, University of Luxembourg, L-4366 Esch-sur-Alzette, Luxembourg; claude.houssemand@uni.lu (C.H.); debora.poncelet@uni.lu (D.P.); christophe.dierendonck@uni.lu (C.D.); 2 Department of Educational Fundamentals and Practices, Université Laval, Québec, QC G1V 0A6, Canada; frederic.guay@fse.ulaval.ca

**Keywords:** social and emotional learning, theory of planned behaviour, contextual variables, teachers’ practices, teachers’ intention

## Abstract

Research has demonstrated that social–emotional learning (SEL) positively influences myriad domains of children’s development. However, the underlying mechanisms influencing teachers’ adoption of SEL remain underexplored. Guided by the Theory of Planned Behaviour (TPB), this quantitative cross-sectional study sought to elucidate the factors that motivate teachers to adopt SEL teaching practices. The study’s sample included 166 volunteer teachers in Luxembourg, recruited as part of a nationwide educational survey. Of these, 82.5% were women. Participants were recruited through convenience sampling, ensuring diversity in socio-economic backgrounds, grade levels, and student needs. Although these findings are based on self-reported data, they offer novel insights by quantifying teachers’ engagement with SEL, with over 50% already implementing related activities. Structural equation modelling shows that the TPB model accounted for 49% of the variance in teachers’ intentions and 44% of the variance in the adoption of SEL practices. Higher intention and self-efficacy predicted more frequent SEL implementation. Teachers with positive SEL attitudes and higher self-efficacy showed greater intention to implement SEL. These findings underscore the significance of cultivating positive attitudes and self-efficacy to facilitate the effective implementation of SEL in educational settings. The role of teacher gender and audience, as well as implications for teaching, professional development, and SEL research, are discussed.

## 1. Introduction

Despite the historical emphasis on academic aptitude within school curricula, the social–emotional learning (SEL) approach has garnered increased prominence in educational settings (Cipriano et al., 2023b; Mahoney et al., 2021; Osher et al., 2016). SEL refers to the social and emotional skills necessary for understanding and regulating emotions, as well as fostering positive relationships and exercising responsible decision-making (CASEL, 2020). The growing enthusiasm for SEL skills has been stimulated by research findings highlighting their relevance in preschool and school settings. Meta-analyses (Cipriano et al., 2023b; Corcoran et al., 2018; Durlak et al., 2011; Kim et al., 2022; Murano et al., 2020; Yang et al., 2019) have shown the importance of SEL programmes in influencing students’ outcomes, including their social and emotional skills, overall well-being, academic performance, and career development.

In the Grand Duchy of Luxembourg, the legislation enacted on 6 February 2009 stipulates that “primary education has the objective of fostering the intellectual, emotional and social capacities of pupils, as well as their ability to discern” (Ministère de l’Éducation Nationale et al., 2009, p. 201). The legislation pertaining to the pedagogy of social and emotional competencies thus confers a degree of autonomy upon educators. It is also noteworthy that the timetable does not specify designated time slots; rather, educators may incorporate these competencies across diverse academic disciplines. For instance, in the context of literacy, students can be encouraged to develop the capacity to comprehend the emotional states of characters depicted in literary works. In the arts, students can learn to recognise and interpret emotions by analysing artworks or expressing emotions through creative activities. It is important to note that these social and emotional skills are not necessarily taught explicitly, and currently, no school textbooks or teaching materials have been distributed in Luxembourg.

A parallel can be drawn between England and Luxembourg in this regard. In both countries, SEL is not a compulsory independent subject, such as mathematics or science, yet aspects of SEL are incorporated into the curriculum. However, it is strongly endorsed and supported by educational programmes and policies. Teachers are provided with evidence-based support materials to implement activities to develop social–emotional skills (Clarke et al., 2015; Goodman et al., 2015). A 2016 study by Wigelsworth et al. revealed that 46% of teachers identified SEL as a priority and 49% considered it to be equally as important as other pressing educational objectives. The study also revealed that almost half of the English teachers questioned said they had devoted more time to SEL over the last five years.

Teachers play a pivotal role in incorporating SEL into classrooms, as they are responsible for deciding whether to incorporate social–emotional competencies into their teaching or to allocate their instructional time to other priorities. However, the role of public policy incentives must also be considered alongside other influencing factors. The objective of this study is to examine the factors influencing teachers’ adoption of SEL practices, including resource allocation, self-confidence, external influence, and attitudes concerning SEL.

### 1.1. Social and Emotional Learning

#### 1.1.1. Overview

In order to explore the motivations that drive teachers to incorporate SEL into practice, it is imperative to provide a comprehensive definition of this construct. According to the Collaborative for Academic, Social, and Emotional Learning (CASEL, 2020), an internationally recognised community of researchers, SEL is defined as “the process through which all young people and adults acquire and apply the knowledge, skills, and attitude to develop healthy identities, manage emotions and achieve personal and collective goals, feel and show empathy for others, establish and maintain supportive relationships, and make responsible and caring decisions” (CASEL, 2020, p. 1). CASEL delineated five core competencies: (a) self-awareness, which encompasses the ability to identify personal strengths and weaknesses, recognise one’s emotions, and understand their influence on behaviour; (b) self-management, which empowers individuals to regulate their emotions, impulses, and behaviour, facilitating progress in life and effective coping with challenges; (c) social awareness, which involves recognizing others’ emotions, considering context, environment, and personal history, comprehending their behaviours and thoughts, and valuing diverse perspectives and backgrounds; (d) relationship skills, which enable effective communication, cooperation, and adaptability, fostering harmonious coexistence, conflict resolution, and cultivating healthy relationships, regardless of individuals’ backgrounds or characteristics; and (e) responsible decision-making, which requires individuals to consider their own and others’ emotions, safety, social norms, and ethical standards in their actions and behaviours.

#### 1.1.2. The Role of Context Variables in Social–Emotional Learning

Several studies have sought to elucidate the influence of specific variables within various SEL frameworks, focusing on the role of gender. Firstly, as Romer et al. (2011) posited, the development of social and emotional competencies differs according to gender. Individuals who identify as female tend to demonstrate higher levels of social and emotional competence. Moreover, R. Evans (2015) underscores the necessity of guaranteeing equitable SEL instruction to prevent the exacerbation of gender disparities. In particular, she highlights the tendency for girls to be expected to demonstrate their emotional competencies more explicitly, whereas boys are frequently encouraged to limit emotional expression, which is interpreted as emotional control. Such differential expectations may, therefore, serve to perpetuate gender stereotypes. Consequently, teachers may unwittingly impede the social and emotional learning opportunities of their male students by adhering to gendered expectations. Furthermore, Collie et al. (2012) demonstrated that gender also influences teachers’ approach to SEL. The results indicate that male educators are more likely to be classified as “SEL-strivers”, a term denoting those with lower comfort levels in delivering SEL content. This finding was confirmed by Molina et al. (2022), who emphasised that female teachers are more inclined to integrate SEL into their classrooms proactively.

Gender is also a pivotal factor in influencing students’ SEL experiences. While girls tend to demonstrate higher competencies, they are subject to heightened expectations. Conversely, boys may encounter obstacles to emotional expression due to the pervasive influence of gender norms. Thus, it is important to evaluate the influence of gender in SEL research. These findings suggest that gender plays a crucial role not only in students’ social and emotional competencies but also in shaping teachers ’attitudes and their implementation of SEL practices. Given these observed differences, it is relevant to examine whether gender influences teachers’ intention and actual adoption of SEL practices, which is discussed in Section 1.2.6.

Nevertheless, additional variables exert an influence on SEL skills, including socio-economic level, as well as age. In the Organization for Economic Co-operation and Development’s (OECD, 2020) report, which examines social and emotional skills, 5-year-olds in Estonia, England and the United States demonstrate age-related growth in emotion attribution and identification skills, regardless of gender, country of origin or socio-economic levels. Additionally, the study revealed that students from more privileged socio-economic backgrounds exhibited superior social and emotional competencies compared to their counterparts from less advantaged socio-economic background. The systematic review and meta-analysis by Cipriano et al. (2023a) corroborate the assertion that students from low socio-economic backgrounds frequently exhibit diminished social and emotional competencies relative to their more affluent counterparts. This discrepancy can be attributed, at least in part, to disparities in access to educational resources and social support. However, given their growth potential, they appear to benefit more from these opportunities (G. W. Evans & English, 2002). The results of these four studies indicate that background characteristics not only influence the specific needs of children, but also their capacity to benefit from social and emotional learning programmes. Social and emotional interventions play a pivotal role in addressing diverse backgrounds and maximizing children’s developmental outcomes. Previous studies have shown that teachers of at-risk children recognise the social and emotional challenges faced by their students and integrate SEL as a strategy to support learning and well-being (Cefai et al., 2018).

Overall, these findings suggest that teachers working with socioeconomically disadvantaged students or those with special needs are more likely to engage in SEL practices to mitigate educational inequities. In the context of students from lower socio-economic backgrounds and those with learning difficulties often exhibiting weaker social and emotional skills, teachers recognise SEL as an essential tool for fostering student development (Cefai et al., 2018; Cipriano et al., 2023a). This body of evidence supports the hypothesis that teachers tailor their engagement with SEL based on the specific needs of their student population.

#### 1.1.3. Effects of Social–Emotional Learning Interventions

SEL offers numerous benefits, both in the short and long term, by equipping students with essential social and emotional skills in conjunction with their academic and cognitive development. Blair and Raver (2015) highlighted a significant correlation between social and emotional competencies and children’s cognitive abilities, mainly executive functions, which are crucial for academic success. Additionally, various meta-analyses have consistently demonstrated that SEL interventions positively influence social–emotional skills, enhance well-being, improve academic performance, and reduce behavioural issues (Blewitt et al., 2018; Kim et al., 2022; Murano et al., 2020; Schindler et al., 2015; Taylor et al., 2017; Yang et al., 2019). For example, the meta-analysis conducted by Durlak et al. in 2011, which encompassed 213 studies involving more than 270,000 students, indicated a weak to moderate influence of SEL interventions on social and emotional skills (effect size (ES) = 0.57), relationship skills (ES = 0.24), emotional distress (ES = 0.24), and academic performance (ES = 0.35). Cipriano et al. (2023b) updated this meta-analysis with 424 studies published between 2008 and 2020 involving 575,361 students and found a positive effect on social and emotional skills, social skills, behaviours, and academic skills, both in the short (overall ES = 0.194) and long term (overall ES = 0.167). Furthermore, they highlighted the advantage of teacher-led delivery on students’ outcomes compared to other school professionals. Additionally, the implementation aspects, such as the sequencing of content and the inclusion of effective programme features (e.g., SAFE practices), played a significant role in the effectiveness of SEL interventions. Furthermore, a meta-analysis (Corcoran et al., 2018) corroborated the effects of SEL interventions on academic performance, specifically in the domains of reading (ES = 0.25), mathematics (ES = 0.26), and science (ES = 0.19). A multitude of meta-analyses have been conducted to evaluate the efficacy of SEL interventions, yielding average effects ranging from 0.19 to 0.38 and effects on behavioural skills ranging from 0.16 to 0.37 (Blewitt et al., 2018; Boncu et al., 2017; Cipriano et al., 2023b; Sabey et al., 2017; Takizawa et al., 2024). Conversely, Moy and Hazen (2018) and Luo et al. (2022) report positive effects on social skills, yet concurrently demonstrate negative effects on behaviour. In this regard, Cook et al. (2015) and Bradshaw et al. (2014) advocate for an intervention that would integrate a behavioural intervention of the Positive Behavioural Interventions and Supports (PBIS) type. These interventions refer to explicit lessons that aim to improve student behaviour.

Moreover, Algan et al. (2022) highlighted the long-term benefits of an SEL programme for children and the society. Their two-year intervention programme aimed to enhance social competencies and self-discipline in children, who were approximately 7 years old at the start of the intervention. Almost thirty years later, they estimated that for each dollar invested, there are 11 dollars in gains for society. Indeed, the programme generates significant economic consequences associated with the decrease in grade repetition, delinquent behaviours, or the utilization of specialised classes, along with an augmentation in taxes attributed to jobs that are well paid. Therefore, SEL seems to have its place in schools. Finally, systematic reviews of systematic reviews corroborated the advantageous short- and long-term outcomes of universal and targeted SEL skills, encompassing both academic and non-academic domains (Durlak et al., 2022; Weare & Nind, 2011; Wigelsworth et al., 2020, 2022).

In Luxembourg, the SEE-learning programme continues to be implemented for classes 2.1 and 3.1 in voluntary schools, with a two-year implementation period. In the United States, a study (Frazier et al., in press) involving 685 children in grades 4 and 5 demonstrated the programme’s effectiveness by documenting the development of intrapersonal constructs over 12 sessions, each lasting approximately 50 min. Additionally, a report for the European Commission by Cefai et al. (2018) identified eight critical elements for the effective implementation of SEL programmes. These elements include consistent programme duration, active participation, early and targeted intervention, and the training and involvement of parents and teachers.

Other researchers in the United States have also disseminated a subset of these recommendations. For instance, Reyes et al. (2012) underscored the significance of teacher training, the frequency of lessons, and the quality of teaching in the implementation of the RULER (recognizing, understanding, labelling, expressing, and regulating) approach, which was developed at Yale University. Dosage was identified as a significant moderator in the meta-analysis by Shi et al. (2022) concerning the effectiveness of the Promoting Alternative Thinking Strategies (PATHS) programme. Their analysis revealed that when the programme was implemented two to three times per week, the effect size was 0.17, but it decreased to 0.03 when the programme was used less frequently. Although SEL interventions appear to be equally effective in Europe, as demonstrated by studies in Luxembourg, a meta-analysis by Sklad et al. (2012) suggests that an SEL intervention loses some of its effectiveness when transposed from its country of origin. This phenomenon was marginally observed in a meta-analysis by Cipriano et al. (2023b) (*p* = 0.08 to *p* = 0.06) but was found in four out of seven studies in a meta-analysis by Wigelsworth et al. in 2016. The authors of the latter study attribute these findings partly to challenges in sharing cultural values and highlight the influence of available resources. They also stress the need for further research to determine the factors that contribute to the effectiveness of SEL interventions.

These findings indicate that SEL interventions are effective in enhancing student well-being and skills, with teachers playing a pivotal role in adapting and implementing them. This raises the question of what motivates teachers to implement SEL practices. According to Kilborn et al. (2024), both knowledge of SEL and the availability of resources significantly influence SEL teaching practices. Furthermore, Zolkoski et al. (2020) demonstrated that self-efficacy and a positive school climate also predict teachers’ intentions to adopt SEL practices. It can therefore be said that various factors shape both the intention and the implementation of these practices.

Several studies have examined ecological models of teacher decision-making focusing on various nano, micro, meso, chrono, and/or macro elements that may influence practices (Horn & Little, 2010; Durlak, 2015; Rimm-Kaufman & Hulleman, 2015). In contrast, this study focuses specifically on the psychological factors that drive teachers to form an intention to adopt a behaviour and subsequently implement it. One theory that elucidates the intrinsic and extrinsic reasons for motivation—particularly within the educational context—is the theory of self-determination (Ryan & Deci, 2017, 2020). According to this theory, the primary motivational factors are autonomy, relatedness, and competence. However, Kupers et al. (2024) employed the TPB and self-determination theory to ascertain teachers’ propensity to discriminate, concluding that while both theories are applicable, the TPB demonstrated a superior alignment with the data. While the self-determination model provides insights into motivation, it does not directly specify the determinants of teachers’ instructional practices. In this context, the Theory of Planned Behaviour (TPB) provides a more appropriate theoretical framework for clarifying the motivations behind the adoption of SEL teaching practices.

### 1.2. The Theory of Planned Behaviour (TPB)

#### 1.2.1. An Overview

**Figure 1:**
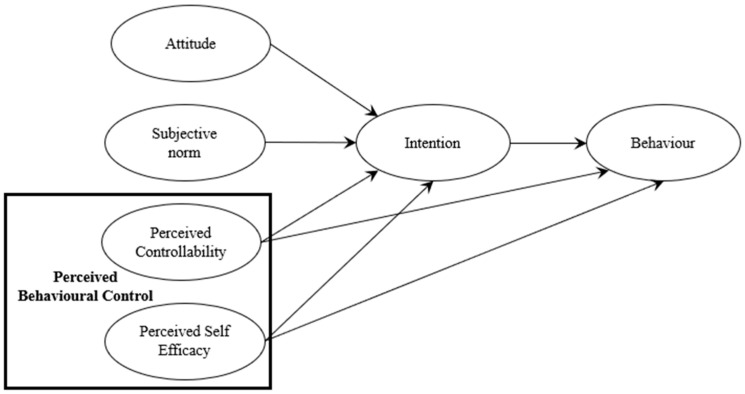
Theory of Planned Behaviour (Ajzen, 1985, 1991, 2002).

As illustrated in Figure 1, the TPB model, developed by Ajzen (1991), aims to predict and understand individual behaviour based on three key components: attitude towards the behaviour, subjective norm and perceived behavioural control. Together, these three factors influence behavioural intention and subsequent actions. This model was selected for this study because it has demonstrated its effectiveness in predicting human intentions and behaviours in various domains such as marketing (Ashaduzzaman et al., 2022), environment and responsible consumption (De Leeuw et al., 2015; Han & Stoel, 2017), health (Rich et al., 2015; Topa & Moriano, 2010), and education (Hellmich et al., 2019; Ingram et al., 2000; Knauder & Koschmieder, 2019; Opoku et al., 2021), but also because other research (Kilborn et al., 2024; Zolkoski et al., 2020) has already shown that the model’s predictor variables, such as attitudes, perceived controllability, and self-efficacy, are part of the explanatory variables for the intention to implement SEL or the reason to teach social–emotional competencies. It seemed interesting to see whether using a model that included all of these variables would have greater explanatory power.

#### 1.2.2. Intention

Ajzen (2019) defined intention as ‘a person’s readiness to perform a given behaviour’. The TPB (Ajzen, 1991) posited that adopting behaviour is contingent upon the presence of intention. Intention also plays a central role since it is predicted by the various variables of interest. Therefore, to understand what motivates teachers to teach SEL skills, it is essential to identify the factors underlying this intention. According to the TPB, intention is influenced by both individual and social factors. Within the individual factor category, teacher attitude towards the behaviour, in this case, the teaching of socio-emotional skills and their self-efficacy, have been shown to impact intention to implement the behaviour. However, this intention is also influenced by external factors, such as subjective norms, which are shaped by the perceptions of those around the respondent, and perceived self-efficacy, which is influenced by the resources available to the respondent. The influence of these variables is moderated by the degree of importance assigned to them, which varies based on the context and the individual. Within the context of SEL, teachers who are convinced of the positive impact of imparting socio-emotional skills to children, who are endorsed by their peers, who feel competent, and who have the necessary resources, are more likely to implement SEL. However, the relative significance of these factors to the teacher will influence their implementation. Consequently, the intention constitutes a focal point of the theoretical framework of TPB, which aims to elucidate the underlying motivations that impel teachers to adopt SEL.

#### 1.2.3. Attitude

The individual factor is intrinsic to an individual’s attitude towards the behaviour in question. It reflects whether the individual perceives adopting such behaviour as leading to positive or negative outcomes, determining its perceived benefits or drawbacks. Eagly and Chaiken (2007) defined attitudes in terms of three key concepts. Firstly, the object of an attitude is that which is being evaluated. Secondly, tendency is based on an individual’s experience. The term is defined as an acquired predisposition to respond positively or negatively to an attitude object. Finally, cognitive evaluation is based on rational, affective, or behavioural beliefs or thoughts. If we apply these three concepts to SEL, an example might be that a teacher might have a positive attitude towards SEL programmes (attitude object) if, from experience, he has the impression that they are beneficial (tendency), because he would feel satisfaction by integrating them into his daily practice (cognitive evaluation). In the TPB (Ajzen, 1991), attitude is identified as a pivotal variable, as it exerts a direct influence on the intention to adopt a given behaviour. Ajzen (2019) represented attitude through a mathematical equation:

“A ∝ ∑ b_i_.e_i_”

He posits that attitude corresponds to the sum of an individual’s beliefs about the outcomes associated with the behaviour in question (b_i_), with the weight assigned to each outcome reflecting the importance the individual attaches to it (e_i_). By way of illustration, in the context of this study, a positive attitude towards SEL means that teachers believe, for example, that SEL has a positive impact on social and emotional skills, classroom climate and academic skills. However, this attitude is weighted according to each of these three areas.

The impact of attitude on intention has been emphasised in studies specifically designed to investigate this relationship, as well as in studies employing the TPB model. To illustrate, Elliott et al. (2015) examined health-risk behaviours, including binge drinking and smoking. The researchers employed three studies to ascertain whether positive and negative attitudes could predict intentions to adopt or not this risky behaviour. Similarly, Petkova et al. (2012) demonstrated that teachers’ attitudes were the most significant predictor of intentions regarding integrating students with physical disabilities into physical education. In the context of TPB, Sheeran et al. (2016) showed in their meta-analysis of 204 experimental tests that interventions aimed at changing attitudes produce significant medium-sized effects on participants’ intentions (d = 0.48). These examples confirm that favourable attitudes play a pivotal role in the formation of intentions, both within and beyond the domain of education. However, this variable is not the sole determinant of intention in the TPB model. Subjective norms also exert a significant influence.

#### 1.2.4. Subjective Norm

The second factor is contingent upon the individual’s sentiments regarding social pressure, particularly regarding the opinions and conduct of significant others. This is referred to as the subjective norm. Ajzen (2019) represents the subjective norm once more through an equation:

“SN ∝ Σ(n_i_.s_i_)”,

where the subjective norm corresponds to the sum of the individual’s beliefs regarding the expectations of significant social referents (n_i_), weighted by the importance the individual ascribes to each referent (s_i_). In the context of our study, if teachers perceive that their colleagues or parents expect them to teach SEL, this may encourage them to implement it. However, this motivation would be weaker compared to a scenario where the expectation comes from a supervisor, whom the teacher may perceive as having a more influential opinion.

In their meta-analysis, Armitage and Conner (2001) corroborated the findings of earlier studies (Sheppard et al., 1988 and Van den Putte, 1991 as cited in Armitage & Conner, 2001) within the TPB model, namely that subjective norm contributes significantly to the prediction of intentions. However, their effect is less pronounced than that of attitude and perceived behavioural control. This relative weakness may be attributed to methodological challenges, such as the lack of sufficiently robust measures and the fact that they often depend on other underlying beliefs. Nevertheless, while subjective norms may be less impactful than attitude or perceived control, they remain a key component in understanding intention formation, particularly in social contexts where peer pressure, institutional policies, or community expectations hold significant sway. It is important to examine whether Luxembourg teachers’ intentions are influenced by their environment.

#### 1.2.5. Perceived Behavioural Control

Ajzen (1985) also introduced a third variable, perceived behavioural control, which is associated with elements influencing intention. On the one hand, perceived behavioural control can be influenced by controlling external factors, such as the availability of sufficient resources or the feasibility of implementing the behaviour. This is what is referred to as perceived controllability. On the other hand, it is also shaped by the perception of one’s own ability to adopt the behaviour. This latter component draws inspiration from Bandura’s (1977, 1982) theory on self-efficacy. Bandura posits that individuals who believe in their capacity to perform a behaviour are more likely to succeed due to their perseverance, compared to those who harbour doubts about their abilities and are more prone to quick abandonment. In the context of SEL, teachers may be swayed by a perception of unfavourable conditions to implement SEL skills or a lack of confidence in their ability to teach these skills. However, studies have called into question the uniqueness of this factor (Armitage & Conner, 1999; Manstead & van Eekelen, 1998; Norman & Hoyle, 2004). Ajzen (2002) clarified that even though perceived self-efficacy and perceived controllability are integral components of perceived behavioural control, it is possible to distinguish the two variables. This is the rationale behind delineating two distinct variables: perceived controllability and perceived self-efficacy. According to the TPB, perceived behavioural control would affect the intention to teach social–emotional skills and the actual adoption of the behaviour. This is because perceived behavioural control incorporates factors external to the individual, which means that even if the intention to adopt the behaviour is present, the availability of resources can significantly influence its effective implementation.

#### 1.2.6. The Influence of Contextual Variables

This commonly used basic model has been supplemented with contextual variables. Ajzen (2005) initially added three sets of background factors: personal (intelligence, emotions, and personality traits), social (age, gender, and race), and informational (experience, knowledge, and media exposure). Despite initially indicating that these variables are not part of the model, he underscored that they could have an impact. Other authors have studied the impact of exogenous variables such as gender or socio-economic level on various factors of the TPB or on adapted TPB models (S. D. Anderson et al., 2017; Dierendonck et al., 2024; Jeffries et al., 2020; O’Connell & Freeney, 2011). For example, as posited by S. D. Anderson et al. (2017), gender influences subjective norms, attitudes and perceived behavioural control about participation in dance lessons outside of school time. Girls exhibit a more favourable attitude toward dance, benefit from a more supportive environment, and perceive fewer barriers to engaging in the activity. These parameters thus explain the greater likelihood of girls participating in dance classes, as postulated by the planned behaviour model. In education, Jeffries et al. (2020) demonstrated that the relationship between gender and the choice of a STEM (science, technology, engineering, and mathematics) subject is mediated by attitude towards science. The study demonstrated that female students tend to exhibit less positive attitudes towards science and are less inclined to pursue STEM subjects than their male counterparts.

From a TPB perspective, these gender-based differences suggest that attitude and perceived behavioural control may mediate the relationship between gender and SEL adoption. Perera et al. (2019) have demonstrated that female teachers tend to exhibit higher levels of confidence in their classroom practices. Kilborn et al. (2024) have demonstrated that teachers who feel more confident in their SEL teaching abilities are more likely to implement SEL practices. Since male teachers report lower self-efficacy in this domain (Collie et al., 2012; Molina et al., 2022), this may partially explain their lower intention to integrate SEL.

It should be noted, however, that gender was not the only factor influencing attitude in this study. Students from higher socio-economic backgrounds exhibit more positive attitudes towards science subjects.

Students’ socio-economic level also influences the variables within the TPB. Dierendonck et al. (2024) used the Theory of Planned Behaviour to show that working with a high proportion of economically disadvantaged students had an impact on attitude, subjective norms and perceived behavioural control. Teachers working with a student population where more than 80% are disadvantaged reported lower perceived behavioural control compared to their counterparts in socially and culturally diverse schools, particularly concerning formative assessment, differentiated instruction, and competency-based practices. Additionally, they exhibited a less positive attitude toward differentiated instruction and competency-based practices than teachers in socially and economically mixed schools, as well as a weaker subjective norm regarding competency-based practices.

These findings suggest that background characteristics, including the type of the population and the gender of the teaching staff, have a significant impact on the variables that constitute the TPB model. This has prompted us to examine the role of these exogenous variables within the model. To the best of our knowledge, no research has been conducted on SEL variables within the TPB model.

#### 1.2.7. The TPB in Education

The TPB model is increasingly employed in education. Erten and Köseoğlu (2022) suggested that this model can be applied not only to the social sciences, health, or marketing context but also to the educational context. They conducted a literature review of 77 articles published between 1990 and 2020 to assess the relevance of the TPB as a model for predicting the behaviour of different actors in education. Their inclusion criteria comprised studies of teachers or students from primary school to university. Their analysis revealed that more than half of the studies identified self-efficacy, perceived controllability, and intention as the key factors influencing behaviour. Dierendonck et al. (2024) demonstrated the applicability of the model in relation to three teacher practices (formative assessment, differentiated instruction, and competency-based practices). MacFarlane and Woolfson (2013) provided evidence that the TPB model can be used in the context of SEL. The objective of the latter study was to identify the factors that influence teachers’ willingness to integrate pupils with social, emotional, and behavioural difficulties in mainstream schools, based on the TPB. The authors identified that subjective norms, particularly the influence of the school principal, play a pivotal role in teachers’ adoption of inclusive practices for these children. The researchers identified attitude and perceived behavioural control as predictors of teachers’ intention to include pupils affected by these disorders. The authors concluded their research by underscoring the value of the TPB model in elucidating the multifaceted and, at times, interrelated factors that shape the adoption of teaching behaviours.

These findings emphasise the TPB model’s efficacy and pervasiveness in elucidating behaviours in educational settings and in identifying the variables that shape teaching practices. Nevertheless, while the TPB has been extensively employed to comprehend teachers’ pedagogical practices, its utilization in the context of SEL remains constrained.

### 1.3. The Present Study

Previous research has shown that SEL interventions have a positive impact on academic, social–emotional, and behavioural skills and well-being outcomes, both in the short and long term (Algan et al., 2022; Corcoran et al., 2018; Durlak et al., 2011; Kim et al., 2022; Murano et al., 2020; Yang et al., 2019). As teachers play a central role in imparting skills, it is essential to investigate whether they integrate SEL into their teaching practices. The TPB is an effective model for understanding why individuals adopt certain behaviours. The main objective of the present study is to determine whether Luxembourg teachers provide SEL to their students and to understand the reasons behind their decision to do so. We hypothesised the following:

- H1. In accordance with the TPB model, the practices related to teaching SEL are directly predicted by perceived controllability, perceived self-efficacy, and intention to teach SEL.
- H2. The intention to engage in SEL is predicted by attitude towards SEL, subjective norms, perceived controllability, and perceived self-efficacy, which are consistent with the TPB model.
- H3. Considering the effects of gender on the different TPB variables in the general population, and more specifically on teachers’ attitude and intention to teach or promote SEL, female teachers show a more positive attitude and greater involvement in SEL than male teachers.
- H4. In order to address the unique needs of children from disadvantaged socio-economic backgrounds, children with special needs, and the youngest children, it is essential that teachers adapt their teaching approaches to meet the needs of their pupils. Consequently, the attitude of the teacher, subjective norm, and perceived behavioural control will be influenced by the audience they teach.

## 2. Materials and Methods

### 2.1. Procedure and Participants

This research was conducted as part of a nationwide survey commissioned by the National Observatory for School Quality (ONQS) and was validated by the Ethics Review Panel of the University of Luxembourg. The aim of this survey was to investigate teachers’ perceptions of their profession and gather their opinions on various reforms concerning the organization of primary education in Luxembourg. Therefore, numerous topics were addressed. This study employs a non-experimental cross-sectional and correlational research design, using a self-report questionnaire to analyse teacher attitudes and practices related to SEL at a single point in time.

In the initial phase, a pre-test of the instrument was conducted by three researchers, and 88 teachers were engaged in May 2021 for a pilot test of the questionnaire, to refine it before teachers were asked to complete it. Teachers were first invited to complete the questionnaire, which enabled us to study the distribution of their responses and the relevance of our questions. Subsequently, ten teachers were orally interviewed regarding the questionnaire’s quality, question clarity, and length. The questionnaire was modified in accordance with the respondents’ input, with the objective of reducing its length. However, given that the questionnaire appraised not only SEL competencies but also the consideration of, and impact from, all of the reforms introduced in Luxembourg in 2009, its length was considerable, with an average completion time of 45 min. The questions pertaining to SEL were situated at the end of the final questionnaire.

In the autumn of 2021, a questionnaire was distributed to each teacher. To avoid the questionnaire becoming excessively protracted, the different cohorts of teachers did not receive the same questionnaire. Regarding the questionnaire containing items on social and emotional skills, 984 pre-school and primary teachers in Luxembourg received an email invitation to respond to the questionnaire administered via Qualtrics. A total of 166 teachers participated in the survey, representing a response rate of 16.9%, suggesting that the sample was probably not representative of the population. Therefore, it is reasonable to conclude that the results could differ at the level of the population. While the sample is based on voluntary participation, it ensures diversity across school levels and socio-economic backgrounds.

On average, respondents had 15.5 years of teaching experience and 82.5% are women. These teachers worked at various educational grade levels: 27.1% in Cycle 1 (for children aged 4 to 5 years), 22.9% in Cycle 2 (6 to 7 years old), 18.7% in Cycle 3 (8 to 9 years old), 21.7% in Cycle 4 (10 to 11 years old), and 9.6% were teachers working across multiple cycles. They also engaged with a diverse audience. Specifically, 12% of teachers worked with classrooms predominantly composed of socio-culturally and economically disadvantaged children. Furthermore, 16.9% of teachers were engaged in the instruction of groups where the majority of students were from low SES backgrounds, 26.5% were responsible for mixed groups with an even distribution of children from both low and high socio-economic levels, 21.7% were working with classrooms where a minority of students faced socio-economic and cultural challenges, and 22.9% were dealing with groups where such children represented only a small fraction of the class. All data were collected while ensuring respondent anonymity.

### 2.2. Measures

The items measuring constructs derived from the TPB were developed for this study to fit the context of SEL.

Attitude was assessed using a set of six newly developed items. These items were inspired by the impact of emotions on academic skills, and the beneficial effects of teaching social–emotional skills, as evidenced by previous research (Audrin, 2020; Brackett et al., 2012; Collie et al., 2012; Pekrun, 2011; Pool et al., 2016; Tornare et al., 2017). Attitude encompasses both affective attitude, allowing judgment of how pleasant it is to adopt a behaviour, with items such as “Developing students’ social-emotional and behavioural skills is satisfying from a professional standpoint”, and instrumental attitude, which measures how beneficial a behaviour is for students, with items like “Developing students’ social-emotional and behavioural skills is useful”. The items were rated on a 6-point Likert scale, ranging from “strongly disagree” to “strongly agree”.

The subjective norm was evaluated using three items that combined descriptive norm, reflecting what significant individuals in the teacher’s environment have accomplished (for example, “The individuals I esteem professionally foster student’s social and emotional and behavioural skills”), and injunctive norm, representing what the teacher perceives as valued by their professional peers (for example, “It is expected of me to develop students’ social-emotional and behavioural skills”). These items were rated using a 6-point Likert scale, ranging from “strongly disagree” to “strongly agree”.

Thirdly, perceived controllability was assessed using four items to verify whether teachers had the resources necessary to implement social and emotional learning activities (for example, “The current conditions in which I work do not allow for the development of students’ social-emotional and behavioural skills”). These items were rated using a Likert scale ranging from “strongly disagree” to “strongly agree”.

To increase the variety of the questionnaire, a cursor was used for the perceived self-efficacy and intention items. This interactive feature mitigated the monotony of a lengthy questionnaire by enabling respondents to adjust a marker along a continuous scale, offering a more dynamic and engaging response mechanism. Perceived self-efficacy was assessed using four items that enabled teachers to express their confidence in teaching various components of social and emotional skills (for example, “Providing effective instruction for emotional learning (helping students recognise and regulate their own and others’ emotions)”). Participants rated their competence on a scale ranging from “not competent at all” to “extremely competent”.

Next, the intention scale consisted of three items (for example, “Implement social-emotional and behavioural skills teaching”) to assess teachers’ commitment to incorporating the teaching of social–emotional skills into their classrooms, using a cursor ranging from “not determined at all” to “fully determined”.

Finally, the behaviour scale was primarily based on elements from the Luxembourg curriculum plan. This scale comprised 10 items (for example, “I teach my students to recognise and regulate their own emotions”). A 7-point scale was used for each item, ranging from “never” to “systematically”.

The exogenous variables comprised grade level (Cycle 1, Cycle 2, Cycle 3, Cycle 4, and two or more cycles, with Cycle 2 as the reference), experience (in years), gender (with female gender as the reference), the school proportion of students with special needs (SSN: more or less than 20%), and the school proportion of socio-culturally and economically disadvantaged children (20% or less, between 20% and 40%, between 40% and 60%, between 60% and 80%, and over 80%, with classes having 40% or fewer disadvantaged students as the reference). These variables were collected at the outset of the questionnaire.

### 2.3. Data Analysis Strategy

A combination of statistical techniques was utilised to test the research hypotheses and assess the robustness of the findings, with all analyses being performed using Mplus 8.3 (Muthén & Muthén, 2017), SPSS 27, and R, specifically the cSEM and MissForest packages.

Adhering to the two-step procedure advocated by J. C. Anderson and Gerbing (1988), the analysis commenced with a confirmatory factor analysis (CFA) to evaluate the quality of the measurement model and the structure of the latent constructs derived from the Theory of Planned Behaviour. Given the ordinal nature of the Likert-type responses, the WLSMV (weighted least squares mean and variance adjusted) estimator was employed. The model fit was assessed using the chi-square statistic (χ^2^), the Comparative Fit Index (CFI), the Tucker–Lewis Index (TLI), the root mean square error of approximation (RMSEA), and the standardized root mean square residual (SRMR), following the recommendations of Hu and Bentler (1999). Model fit was considered adequate if the CFI and TLI values were equal to or above 0.90, and the RMSEA and SRMR values were below 0.08. A statistical significance threshold of *p* < 0.05 was employed.

Following the CFA, structural equation modelling (SEM) was employed to assess the hypothesized relationships between the latent variables (attitude, subjective norm, perceived behavioural control, intention, and behaviour) as postulated by the TPB. This step enabled the empirical testing of the model’s core assumptions about the interaction of these constructs in explaining teachers’ SEL-related intentions and behaviours.

However, convergence issues in Mplus, arising from the absence of responses for specific categorical options, precluded the direct integration of exogenous variables such as gender, years of experience, grade level, and school context into the SEM model. To address this issue, latent factor scores were computed in Mplus and subsequently imported into SPSS for multiple regression analyses, enabling the exploration of the influence of these background characteristics on the TPB constructs.

In order to enhance the robustness of the findings, a complementary analysis was performed using partial least squares structural equation modelling (PLS-SEM) with the cSEM package in R. This method, recommended by Hair et al. (2017), is particularly suitable for small to medium sample sizes and emphasises predictive accuracy over model fit. To ensure the integrity of the dataset utilised for PLS-SEM, missing values were imputed using the MissForest algorithm, a non-parametric technique based on random forest models.

Subsequently, multicollinearity was assessed by computing variance inflation factors (VIF), which all remained below the critical threshold of 5, indicating acceptable levels of collinearity among predictors. Additionally, R^2^ values were reported to evaluate the proportion of variance explained in the intention and behaviour constructs. To ensure adequate statistical power, a priori sample size estimation was conducted using the inverse square root method (Kock & Hadaya, 2018), based on a minimum expected path coefficient of β = 0.23. The results indicated that a minimum of 117 participants would be needed to detect such an effect with 80% power at a significance level of 5%, confirming that the 166 participants in this study constituted a satisfactory sample size.

## 3. Results

This section presents the results in alignment with the research objectives and hypotheses. Initially, descriptive analyses were conducted to provide a comprehensive overview of teachers’ attitudes, perceived self-efficacy, and the implementation of SEL practices.

The internal consistency of the scales was evaluated using composite reliability (CR) in accordance with Wang and Wang’s (2020) recommendations. CR was 0.875 for the attitude scale, 0.636 for the subjective norm scale, 0.704 for the perceived controllability scale, 0.925 for the perceived self-efficacy scale, 0.913 for the intention scale, and 0.924 for the behaviour scale.

Subsequently, confirmatory factor analysis (CFA) was conducted to ascertain the measurement model’s reliability before the hypothesis testing. SEM was then used to assess the predictive relationships outlined in the TPB framework, particularly in testing H1 and H2. Finally, regression analyses examined the influence of gender (H3) and student characteristics (H4) on teachers’ attitudes, perceived control, and SEL engagement.

### 3.1. Descriptive Analyses

The items’ descriptive statistics for each variable in the TPB model are presented in Appendix A. The results indicated that over 75% of the surveyed teachers exhibited response patterns suggesting their professional satisfaction and agreement regarding the benefits of SEL, both for students and themselves. Concerning the items employed to assess the subjective norm variable applied to SEL, most teachers believed that this activity is expected and already practiced by colleagues they regarded as professionally competent. The data also indicated that a significant number of teachers expressed confidence in their ability to teach social and emotional skills despite some variability in the responses. As for perceived controllability, the results reveal that more than two-thirds of the surveyed teachers considered that current conditions do not appear to hinder teachers’ willingness to propose SEL. Nevertheless, almost half of the teachers indicated a lack of sufficient resources to implement SEL.

Given the elevated attitude, subjective norms, perceived self-efficacy and perceived controllability, and given the supposition of both H1 and H2, it was anticipated that the intention to teach SEL and SEL practices will also be elevated. This assertion was further substantiated by Appendix A, which revealed a relatively strong intention among teachers to implement SEL, although this intention is subject to variability in the responses. Regarding SEL practices, the data demonstrate that teachers are implementing at least some SEL skills. For instance, over 60% of teachers indicated that they “frequently” teach the regulation and recognition of their own emotions and behaviours, as well as the recognition of other people’s emotions.

However, the surveyed teachers reported that social and emotional skills were predominantly addressed reactively rather than proactively in instances where issues arose. Indeed, in response to the item “I organise social and emotional learning activities”, teachers predominantly selected responses ranging from “sometimes” to “frequently”, while more than half of the teachers opted for responses ranging from “frequently” to “systematically” when it came to leveraging an event (items beh6, beh7, beh8, and beh9) to work on social and emotional skills.

### 3.2. Measurement Model

**Figure 2:**
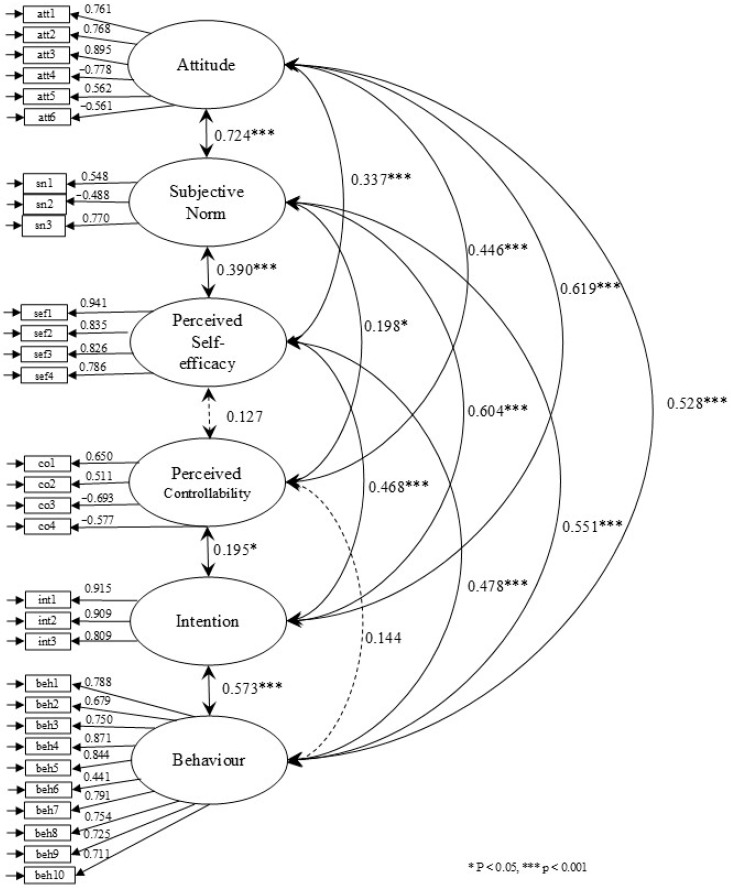
Confirmatory factor model for the TPB applied to SEL.

The measurement model is shown in Figure 2. The model demonstrated a good fit with the data (χ^2^ = 3586.784, df = 435, *p* < 0.001, CFI = 0.914, TLI = 0.904, RMSEA = 0.065, SRMR = 0.070). The items loading on their latent factor were significant and high (>0.400), thus allowing for further analyses.

### 3.3. Predicting Intention and SEL Practices (H1 and H2)

**Figure 3:**
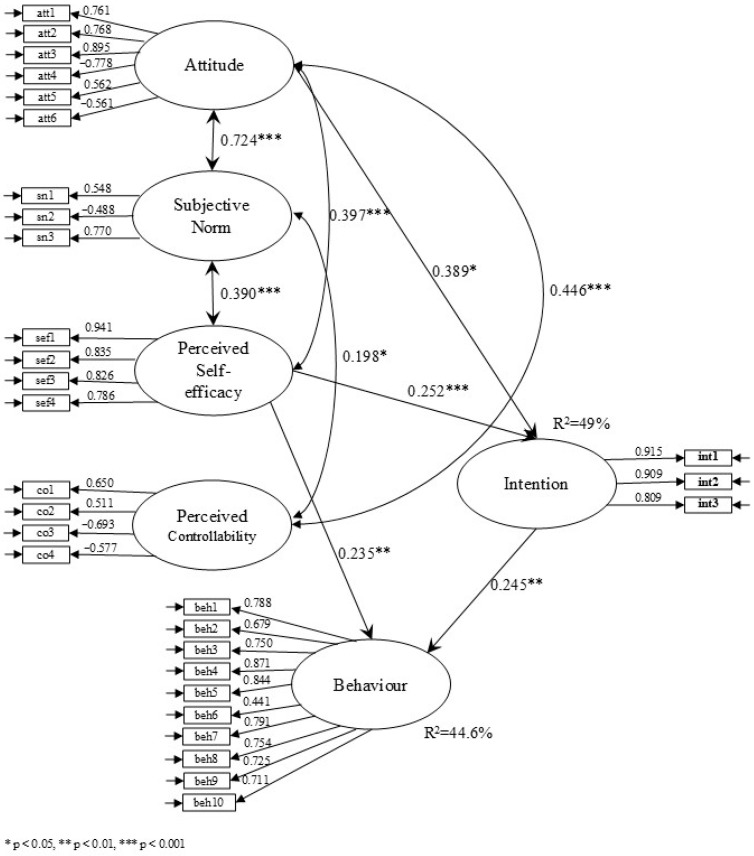
Structural equation model based on the TPB and aiming to explain the use of SEL.

The results of structural equation modelling (Figure 3), which included both the measurement and structural model, demonstrated a good fit to the observed data (χ^2^ = 662.315, df = 390, *p* < 0.001, CFI = 0.914, TLI = 0.904, RMSEA = 0.065, SRMR = 0.070). For more clarity, non-significant links were not displayed in the figure below.

According to Figure 3, variables measuring attitude, subjective norm, perceived self-efficacy, and perceived controllability exhibited significant correlations (r = 0.724, r = 0.390, r = 0.397, r = 0.198, r = 0.446). However, the correlation between perceived self-efficacy and perceived controllability was not significant. This suggests that teachers’ perceptions of available resources are not directly related to their feelings of self-efficacy.

Of the original four predictive variables in the model, only attitude and perceived self-efficacy were significantly and positively associated with the intention to implement SEL practices. A high intention to teach SEL was associated with a more favourable attitude towards SEL (β = 0.389) and a more positive perceived self-efficacy (β = 0.252). Frequent SEL practices, on the other hand, were associated with high perceived self-efficacy (β = 0.235) and high intention (β = 0.245). In other words, the more teachers expressed the intention to adopt the behaviour, the more inclined they were to put it into practice effectively. Moreover, the more competent they felt to do so, the more likely they were to implement it.

The Theory of Planned Behaviour model applied to the teaching of social and emotional skills explained 49% of the variance in teachers’ intention to implement SEL and 44.6% of the variance in SEL practices.

A supplementary analysis utilising partial least squares (PLS) was conducted to evaluate the extent of multicollinearity between the independent variables within the model. This was achieved by calculating the variance inflation factor (VIF). This supplementary analysis, which corroborates our initial findings, showed that all VIF values for the independent variables were below the critical threshold of five, with some, including perceived self-efficacy, attitude, perceived controllability, and subjective norm in relation to intention, even falling below two. This indicates a low level of multicollinearity. These results corroborate the satisfactory interpretability of the independent variable coefficients and the reliability of the regression estimates. The quality of the prediction is further validated by the following R^2^ values: intentions (0.56; f^2^: 1.272) and practices (0.48; f^2^: 0.93).

### 3.4. Influence of Gender and Contextual Variables (H3 and H4)

To assess the role of contextual variables in the TPB model’s variables, regression analyses were conducted using four exogenous variables (Table 1). Factor scores were generated from the Mplus measurement model and imported into SPSS to conduct multiple regression analyses.

The results presented in Table 1 indicate that the grade level had an influence on behaviour (β = 0.20), attitude (β = 0.26), subjective norm (β = 0.27), and perceived controllability (β = 0.24). Compared to Cycle 2, teachers in Cycle 1 demonstrated a more positive attitude towards SEL, as well as a higher frequency of implementation or encouragement to implement SEL by colleagues and superiors. They also exhibited an increased sense of having the necessary resources to implement SEL. The predictive variable SSN revealed that, compared to teachers working in schools with 20% or fewer SSN, those in schools with more than 20% SSN had a more positive attitude towards SEL (β = 0.17), a higher frequency of implementation or encouragement to implement SEL by colleagues and superiors (β = 0.24), and more frequent implementation of SEL by the teachers themselves (β = 0.16). Moreover, gender predicted teachers’ attitude (β = −0.22). Male teachers showed a less favourable attitude towards SEL than their female counterparts. Finally, teachers working in disadvantaged schools with 60 to 80% socio-cultural and economic disadvantage perceived themselves to have enough resources for SEL implementation (β = 0.22), compared to those working in privileged schools with 40% or fewer students. Conversely, teachers in favoured schools with less than 20% socio-culturally and economically disadvantaged students had a lesser sense that they had sufficient resources (β = −0.12). No exogenous variables introduced seemed to influence perceived self-efficacy. All of these results are in line not only with our hypotheses but also with other studies. These results provide valuable insights into the factors influencing SEL integration. The implications of these results for interventions are discussed in the following sections.

## 4. Discussion

The objective of this study was twofold: firstly, to comprehend teachers’ perspectives on SEL, and secondly, to ascertain the motivating factors that influence their implementation of these practices or their intention to do so. To this end, we drew upon Ajzen’s (1991) TPB, which posits that the adoption of SEL is influenced not only by perceived behavioural control but also by the intention to adopt this behaviour. This intention is then influenced by subjective norms, attitude, and perceived behavioural control, which are defined by self-efficacy and perceived controllability. Each of these dimensions was assessed through specific items, which also offered valuable insights into the stances and perceptions of teachers on each scale.

### 4.1. The Theory of Planned Behaviour Applied to Social and Emotional Learning

The study’s findings support the relevance of part of the TPB model in predicting the intention and teaching practices related to SEL. According to our study, the model effectively predicts intention (49% explained variance) and behaviours (44.6% explained variance). The association between attitude, subjective norms, perceived self-efficacy and perceived controllability (Figure 3) highlights the need for a holistic approach that considers these interrelated dimensions to successfully implement SEL in the school context. Nevertheless, it is important to consider a more nuanced interpretation of our initial first two hypotheses.

**H1.**  *In accordance with the TPB model, the practices related to teaching SEL are directly predicted by perceived controllability, perceived self-efficacy, and intention to teach SEL.*

According to TPB, teachers’ intention, perceived controllability, and perceived self-efficacy directly predict their practices regarding their teaching of social and emotional skills. In our study (Figure 3), intention and perceived self-efficacy effectively predict teachers’ behaviour concerning SEL practices. However, perceived controllability has no significant effect on behaviour. Terry and O’Leary (1995) posit that, given the tendency for perceived self-efficacy and perceived controllability to be amalgamated within perceived behavioural control, earlier measures of perceived behavioural control may have predominantly captured perceived self-efficacy effects. This hypothesis offers a potential explanation for the previously observed correlations between perceived behavioural control and intentions. Another hypothesis is that the lack of resources as indicated by nearly half of the respondents (Table A1) may have been a contributing factor. Conversely, the findings of this study demonstrate the pivotal role of perceived self-efficacy and intentions in the adoption of novel classroom practices.

However, Sniehotta et al. (2014) emphasise the limitations of TPB. They argue that, although the TPB offers insight into the psychological predictors of SEL adoption, it does not always account for other factors that may directly influence behaviour, independently of stated intentions. Consequently, while the present study underscores the pivotal roles of attitude and self-efficacy in the intention to teach SEL, Sniehotta et al. (2014) identify alternative determinants, including habit strength, motivational factors such as self-determination, anticipated regret, and nudging. These factors pertain to more subtle changes in teachers’ practices, which may be imperceptible to the teacher, such as the addition of tools in the classroom to support SEL. These various determinants could be the subject of further research to achieve a more comprehensive understanding of the factors that motivate teachers to implement SEL in their practice.

**H2.**  *The intention to engage in SEL is predicted by attitude towards SEL, subjective norms, perceived controllability, and perceived self-efficacy, which are consistent with the TPB model.*

In Ajzen’s TPB model, intentions are also directly predicted by perceived controllability, self-efficacy, subjective norms, and teachers’ attitudes. Although attitude and self-efficacy are predictive of intention, subjective norms and perceived controllability, in our model, do not play a significant role. This aligns with Manstead and Van Eekelen’s (1998) study, where subjective norms and perceived controllability are not systematically predictive of intention and behaviours. In relation to the concept of perceived controllability, the theory that teachers possess the necessary skills to create their own resources and are less dependent on external resources is also applicable to intentions. In their meta-analysis, Armitage and Conner (2001) corroborate the findings of earlier studies (Sheppard et al., 1988 and Van den Putte, 1991, cited in Armitage & Conner, 2001) within the TPB model. Specifically, they demonstrate that, while subjective norms contribute significantly to the prediction of intentions, their effect is less pronounced than that of attitude and perceived behavioural control. According to the authors, this relative weakness may be attributed to methodological challenges, such as the lack of sufficiently robust measures and the fact that they are often dependent on other underlying beliefs. Indeed, it can be assumed that it is sometimes complex to obtain reliable data when it comes to self-declared responses concerning opinions attributed to others.

Furthermore, the social and emotional skills are not taught in isolation but in conjunction with other academic skills, such as expressing emotions through theatrical performances or the ability to empathise with characters in literary texts (Ministère de l’Éducation Nationale & de l’Enfance et de la Jeunesse, 2011). It is possible that teachers may perceive these skills more as a recommendation rather than a compulsory requirement.

This hypothesis is supported by our analysis of teacher behaviours (Table A1), which indicates that teachers are more inclined to incorporate SEL in response to specific challenges rather than proactively. A forthcoming study will explore whether colleagues and headteachers recognise the importance of teaching socio-emotional skills. Should this not be the case, it could be indicative of underdeveloped social expectations regarding SEL instruction, potentially explaining the ineffectiveness of the subjective norms scale in this context.

Furthermore, the findings of this study indicate that perceived controllability did not significantly predict SEL practices. Teachers generally reported a high degree of perceived control over SEL implementation (Table A1). This homogeneity may have reduced its ability to differentiate between those who do and do not engage in SEL practices, thereby limiting its predictive strength in the model. According to Sheeran et al. (2003), perceived behavioural control is a strong predictor of behaviour only when individuals accurately assess their own ability to perform the action. Should teachers in our study have overestimated their capacity to implement SEL, this could explain why perceived controllability was not significantly related to behaviour. To gain a more comprehensive understanding of the relationship between PBC and SEL implementation, future research could incorporate direct measures of actual control, such as classroom observations or teacher interviews.

In essence, it can be concluded that attitude and self-efficacy are the key variables influencing teachers’ intentions and, consequently, the adoption of SEL practices. Alsalamah’s (2023) findings indicate that teachers who have participated in more professional development training exhibit a higher level of self-efficacy. Furthermore, Perera et al. (2019) propose a taxonomy of self-efficacy profiles, and posit that mentoring and professional training, when coupled with practical application, are instrumental in fostering self-efficacy. The findings of our study demonstrate the importance of providing educators with information regarding the advantages of integrating social and emotional skills into their instructional practice, with reference to the favourable impact on the learning outcomes of their pupils to enhance teachers’ attitudes towards SEL, as well as methodological contributions to the three components of SEL, i.e., emotional, social, and behavioural skills, to increase teachers’ self-efficacy. This finding is consistent with the results reported by Shi and Cheung (2024) in their meta-analysis. Indeed, the authors indicate that a focus on socio-emotional skills during training significantly enhances the efficacy of SEL interventions, as evidenced by an effect size of ES = 0.10, *p* < 0.05. In their respective contributions, the study by Duane et al. (2025) underscores the pivotal function that university-based continuing education programmes focused on SEL can fulfil. Following participation in a training course, educators reported not only an augmentation in SEL-related competencies but also a substantial enhancement in their perception of institutional support. These two components, perceived competence and perceived support, are recognised as indispensable prerequisites for the effective implementation of SEL in the classroom. This study underscores the significance of developing high-quality continuing education opportunities to bolster teachers’ sense of self-efficacy and, consequently, to augment their intention to integrate SEL into their pedagogical practices.

In accordance with the TPB model, such interventions would be expected to increase teachers’ intentions to teach SEL and their actual implementation of SEL practices, thereby facilitating the development of children’s well-being and academic and socio-emotional skills, for which there is a robust corpus of evidence (Cipriano et al., 2023b; Durlak et al., 2022; Wigelsworth et al., 2020, 2022).

### 4.2. Teacher Positioning for SEL and the Influence of Contextual Variables

According to our results (Appendix A), teachers exhibit a generally positive attitude towards instruction on social–emotional and behavioural skills. They seem to acknowledge the positive effects of SEL, as demonstrated by various researchers, including its contribution to academic achievement, the development of social–emotional and behavioural skills, and overall well-being (Boncu et al., 2017; Blewitt et al., 2018; Charlton et al., 2021; Collie et al., 2012; Collie, 2020; Corcoran et al., 2018; Durlak et al., 2011; Grant et al., 2017; Wigelsworth et al., 2016). Nevertheless, it would be worthwhile to undertake a more in-depth analysis of these results and ascertain whether exogenous variables exerted an influence on the TPB variables.

**H3.**  *Considering the effects of gender on the different TPB variables on the general population, and more specifically on teachers’ attitude and intention to teach or promote SEL, female teachers show a more positive attitude and greater involvement in SEL than male teachers.*

The findings of this study suggest that gender is a predictor of teachers’ attitudes towards SEL, with male teachers exhibiting less favourable attitudes compared to their female counterparts (β = −0.22). This assertion is corroborated by extant studies. For instance, a survey in the USA found that female teachers were 14 percent more likely than their male colleagues to rate SEL as ‘very important’ or ‘essential’ in the school (Rikoon et al., 2024). Similarly, in Australia, Molina et al. (2022) found that female teachers, particularly in primary schools, were more supportive of the benefits of SEL and more likely to advocate its integration into their teaching practices. These results suggest that differences in attitude are not anecdotal but instead reflect underlying socio-cultural and institutional dynamics.

The divergent attitudes towards SEL among male and female teachers can be attributed to several socio-cultural and institutional factors. Gender stereotyping has been shown to play a pivotal role from an early age. In numerous cultures, male children are encouraged to suppress their emotions, while female children are more encouraged to express their feelings and demonstrate empathy (Chaplin, 2015). As a result, female teachers may find it more natural to incorporate SEL into their teaching practices, since they have internalised these norms from a young age. In contrast, their male counterparts may have internalised the belief that SEL is not their priority, reinforcing their reluctance to engage with such practices. In a recent study, Avissar (2023) examined the impact of emergency distance teaching on the social–emotional connection between teachers and students. The study revealed that male teachers reported a more significant deterioration in their social–emotional connection with students compared to their female counterparts. These findings suggest that male teachers may face challenges in adapting to the relational and emotional aspects of teaching, particularly in challenging or unfamiliar contexts. These findings underscore the necessity for tailored support and targeted SEL training that addresses these gender-related perceptions and readiness.

However, a study by Shahat et al. (2022) challenges this notion, suggesting that male teachers have more positive attitudes towards SEL than female teachers. The higher proportion of male teachers in their sample and the increased likelihood of male teachers attending training courses on social and emotional learning are cited as justifications for this discrepancy. The findings suggest that initial and in-service training may substantially impact teachers’ attitudes towards these competencies. These observations suggest that the absence of targeted training may contribute to the reluctance of some teachers, particularly male teachers, to become involved in teaching SEL.

Considering these findings, several strategies can be implemented to reduce the observed differences in attitude between teachers and encourage greater involvement of male teachers in SEL. A fundamental lever for change is the enhancement of initial and in-service training (Shahat et al., 2022). The highlighting of male role models involved in SEL can also help to change the perception that this is an exclusively ‘female’ approach to education. Furthermore, it is crucial to deconstruct gender stereotypes within educational institutions. One approach is the provision of spaces for discussion, where teachers can critically examine traditional norms associated with their profession and reflect on their own relationship with social and emotional learning. The next step is to raise awareness of institutional gender stereotypes as barriers to the adoption of these competencies. Addressing these biases can foster a more inclusive approach to SEL integration in educational settings (R. Evans, 2015; Molina et al., 2022). Finally, highlighting the concrete benefits of social and emotional learning—particularly its capacity to enhance student behaviour and academic performance—can be instrumental in persuading teachers who may initially be reluctant to adopt these practices. In conclusion, while male teachers tend to exhibit less favourable attitudes towards SEL, this disparity can be addressed. Targeted interventions focusing on teacher training, institutional reform, and the demonstration of SEL’s practical benefits can encourage broader engagement with SEL among teachers.

**H4.**  *In order to address the unique needs of children from disadvantaged socio-economic backgrounds, children with special needs, and the youngest children, it is essential that teachers adapt their teaching approaches to meet the needs of their pupils. Consequently, the attitude of the teacher, subjective norm, and perceived behavioural control will be influenced by the audience they teach.*

As hypothesised, the greater the number of students with special needs taught by a given teacher, the more positive their attitude towards SEL, and the more likely they are to have colleagues or superiors who implement or recommend these practices and the more these practices are implemented (Table 1). The results of this study indicate that the propensity towards SEL, in terms of attitude, subjective norm, and teacher practices when confronted with a greater proportion of students with special needs, may stem from the perception that these social and emotional skills offer a pertinent response to address daily challenges.

Urton et al. (2023) conducted a study applying the TPB to inclusive teaching practices in German primary schools. The study found that teachers’ intentions were strongly influenced by psychosocial variables, including attitude, subjective norm, and perceived behavioural control. Although the study did not specifically assess SEL, inclusive practices often rely on strong socio-emotional competencies. This finding contrasts with our own, where subjective norm and perceived controllability were not significant predictors. One potential explanation for this discrepancy could be that, in our sample, these constructs were less salient or less effectively activated. Alternatively, it may reflect a difference in how shared norms and actual control are perceived across contexts. This highlights the importance of further investigating how psychosocial predictors interact with specific teaching populations and institutional environments when implementing SEL.

Murano et al. (2020) suggested in their meta-analysis that studies demonstrate the positive effect of SEL interventions on at-risk students. Proposing targeted interventions for small groups of students experiencing social and emotional difficulties would be an interesting approach to providing early support. Moreover, they argue that, considering the critical period of early childhood for children’s development, the implementation of universal SEL interventions in preschool should demonstrate significant advantages.

A study conducted across five European countries has demonstrated that in-service training programmes in SEL equip teachers with strategies to mitigate behavioural issues among pupils (Berg et al., 2021). Teachers who have undergone SEL training report greater levels of comfort and confidence in integrating these competencies into their pedagogical practices, thereby enhancing their willingness to implement such strategies in the classroom. In contrast, when teachers receive no training, they express a greater need for support. For instance, a survey conducted by Chow and Sharma (2022) in Hong Kong revealed that on average, teachers felt inadequately trained and supported for inclusive practices, particularly when dealing with pupils exhibiting social or behavioural difficulties. In this study, the level of teacher training and experience with pupils with special needs emerged as significant predictors of their perceived need for support. The findings suggest that the more training teachers received in SEL and inclusive education, the more confident and better equipped they felt. These results corroborate the idea that teachers require additional training in socio-emotional management to effectively address the diverse needs of their class, mainly when teaching pupils with special needs.

Moreover, the institutional context plays a crucial role. Research indicates that the quality of SEL implementation depends heavily on factors linked to the school and the support available to teachers (Humphrey, 2013; MacFarlane & Woolfson, 2013). Luxembourg has notably increased the resources allocated to pupils with special needs, demonstrating a strong institutional commitment to inclusion. (Ministère de l’Éducation Nationale & de l’Enfance et de la Jeunesse, 2022) This proactive policy is accompanied by a growing recognition of the importance of socio-emotional skills. A national survey emphasises that enhancing socio-emotional skills and fostering positive relationships in the classroom are essential for pupil success and well-being, and advocates for a greater integration of SEL into teacher training (ONQS, 2024). In practice, teachers in Luxembourg increasingly benefit from additional support, such as support teams for pupils with educational needs and the presence of specialised teachers in primary education. These measures can alleviate the challenges associated with managing pupils with special needs. Nevertheless, as in many other countries, teachers still face the challenge of adapting their socio-emotional teaching to highly diverse classrooms. Institutional efforts, including the 2023 law on pupil welfare and inclusive education, are specifically designed to provide greater support to teachers. Educators encountering socio-emotional difficulties in their classrooms can access specialised training through the Centre for Social and Emotional Development. It can therefore be said that, in Luxembourg, as elsewhere, teachers with a higher number of pupils with special needs tend to invest more in SEL, supported by additional training and nationally provided resources.

The findings of this study indicated that the grade level emerges as a predictor of several variables within the TPB model, namely attitude, subjective norm, behaviour, and perceived controllability. Perhaps at this school level, the teaching of socio-emotional skills coincides most closely with the school curriculum. Indeed, Cycle 1 is often designed to enable children to socialise and begin learning to adapt their behaviour to their new environment, and teaching socio-emotional skills can help teachers achieve this goal. Concerning the subjective norm, respondents participating in the questionnaire appear to predominantly operate within an environment where interventions related to social–emotional skills are commonplace and endorsed by school management (Appendix A). These outcomes align with the research by MacFarlane and Woolfson (2013), which highlighted the importance of school leaders in including students with challenges within mainstream educational institutions.

Finally, despite the limited number of significant results regarding the impact of students’ socio-economic status, it was observed that teachers exhibited a reduced propensity to teach SEL when confronted with an audience of a higher socio-economic status (Table 1). This phenomenon may be attributed to the documented finding that students from lower socio-economic backgrounds exhibit comparatively diminished socio-emotional skills in comparison to their counterparts from higher socio-economic backgrounds (Cipriano et al., 2023a; Gruijters et al., 2023; OECD, 2020). This observation supports the hypothesis that teachers working with children from higher socio-economic backgrounds may feel less obliged to incorporate SEL into their pedagogical practices than their counterparts working with students from lower socio-economic backgrounds.

A pivotal factor relates to the expectations of both the education system and parents. Institutions catering to affluent demographics may often be subject to heightened expectations as reflected in examinations, selective guidance, and other performance metrics. This academic pressure can lead to the marginalisation of SEL.

A survey of American teachers found that approximately one-third of respondents felt constrained by academic objectives, to the extent that they were unable to allocate sufficient time to their students’ socio-emotional development (Kurtz et al., 2019). Moreover, 27% of respondents reported a lack of support from families when attempting to incorporate these skills into their teaching practices. Luxembourg, a country with a high standard of living, offers a particularly relevant case study in this regard. While only a limited number of peer-reviewed studies have examined SEL implementation by socio-economic background in Luxembourg, the national education system is progressively acknowledging the significance of socio-emotional skills for all pupils (Ministère de l’Éducation Nationale & de l’Enfance et de la Jeunesse, 2022). It is also noteworthy that Luxembourg has a highly diverse school population, with 44.7% of pupils holding foreign nationality. Furthermore, the school utilises three languages in its instructional practices (Ministère de l’Éducation Nationale et al., 2024). This multicultural and multilingual context presents unique socio-emotional challenges (e.g., integration and cultural adaptation) that are not necessarily linked to socio-economic status. Taylor et al.’s (2017) meta-analysis demonstrates the efficacy of SEL teaching for all students, irrespective of socio-economic status. Acknowledging the common ground between affluent and non-affluent students who benefit from intentional social–emotional teaching is pivotal. Instead of competing with academic goals, SEL methods should be adapted to align with institutional priorities. Understanding these dynamics is crucial for refining teacher training and school policies, ensuring that SEL is not perceived as a luxury reserved for pupils in difficulty but as a fundamental component of education for all.

Thus, in our study, the TPB model variables effectively predict intentions and behaviours related to SEL, even though subjective norms and perceived behavioural control do not predict intentions. Various TPB variables are influenced by contextual factors. Teachers are influenced by their audience (the number of SSN, children’s SES, and grade level) and their gender. This study also found that teachers already integrate activities to develop SEL skills. However, the pedagogical approach primarily occurs in response to problematic social and emotional situations encountered in students’ daily lives, rather than through dedicated activities preceding such situations. This is a departure from what might be expected, which is the prior integration of specific activities aimed at promoting the development of social and emotional skills in all students, aiming to avoid problems before they happen, rather than solve them.

### 4.3. Limitations

Although our results correspond to, or even exceed, the expected results of the TPB model, recording 49% and 44.6% compared to the 39% and 27% of variance explained in the intention and practices of the meta-analysis of Armitage and Conner in 2001, certain limitations inherent to this study should be considered.

It is important to note that due to the relatively small size of the sample, it is possible that only those teachers who were particularly motivated to participate in the survey may have done so, which could have an impact on the representativity of our sample and on the generalization of the study results to the national population of elementary teachers.

Furthermore, while the cross-sectional nature of the data remains appropriate for identifying associations and generating hypotheses, as all of the data were collected at a single point in time, the insights gained into the associations between these variables provide a strong foundation for further exploration. However, these findings should be tested in a longitudinal study at a later stage.

While this study employed both covariance-based structural equation modelling (SEM) and partial least squares SEM (PLS-SEM) to assess the robustness of the model, other advanced analytical approaches could be considered in future research. For instance, Bayesian SEM has the capacity to accommodate the incorporation of prior distributions, and multilevel SEM would be particularly well-suited to data structures involving nested levels (e.g., students within classrooms). Additionally, the utilization of longitudinal data would facilitate the assessment of causal pathways over time, a capability that is not feasible within the confines of the current cross-sectional design.

Furthermore, despite clear explanations about the assurance of anonymity and the importance of providing truthful responses, the possibility of a response bias, inherent in respondents’ desire to conform to presumed expectations, must be considered. Since the answers are based on self-reporting, the results could be distorted. Ideally, the direct observation of teachers’ practices would have provided a more objective approach.

These limitations highlight the importance of examining our conclusions with caution and encouraging further research based on alternative or more in-depth methodologies to better understand the implementation of SEL in an educational context.

## 5. Conclusions

The present study set out to ascertain the psychological and contextual factors that exert influence on teachers’ implementation of SEL practices. Utilising the TPB, the study’s findings confirmed that attitude and perceived self-efficacy are pivotal in predicting teachers’ intentions, which in turn influence their classroom behaviours. However, other TPB variables such as the subjective norm and perceived controllability were not significant in the model developed, prompting further investigation into the operationalisation and perception of these constructs in diverse teaching contexts. The study also underscores the role of contextual factors, such as grade level and the proportion of students with special educational needs, in shaping teachers’ engagement with SEL. Notably, gender differences emerged in attitudes towards SEL, with male teachers expressing less favourable views, an observation supported by recent literature and pointing to the importance of tailored professional development.

However, this study has several limitations. Firstly, the use of self-reported data may have introduced social desirability bias, particularly regarding sensitive constructs like attitudes and norms. Secondly, while efforts were made to ensure anonymity and encourage honest responses, the absence of behavioural observation limits the objectivity of the data. The cross-sectional design restricts causal interpretation, and longitudinal approaches combined with qualitative or observational methods would be beneficial for future studies to capture the evolving nature of SEL practices and teacher motivation over time.

From a practical standpoint, the findings emphasise the necessity to strengthen in-service training programmes that provide concrete tools for SEL instruction and actively foster teachers’ self-efficacy and positive beliefs about SEL’s value. Furthermore, aligning SEL with existing curricular objectives could ease the perceived burden and support its integration as a core educational component rather than a peripheral add-on. As the literature suggests (e.g., Duane et al., 2025; Alsalamah, 2023; Shi & Cheung, 2024), training that combines emotional skill-building, institutional support, and opportunities for reflective practice is especially effective in enhancing SEL implementation.

Finally, although the TPB provided a relevant framework for identifying the psychological determinants of SEL implementation, the results of the present study also suggest the model’s limitations in fully capturing the complexity of teachers’ engagement. Constructs such as perceived institutional support, emotional exhaustion, or habitual teaching patterns, absent from the TPB, appear to play a meaningful role in shaping SEL practices, as highlighted in recent empirical studies. This finding calls for the enrichment of the model through the incorporation of additional psychosocial dimensions that are more suited to the institutional and cultural realities of schools. Future research could explore these extensions, drawing from ecological, motivational, or behavioural frameworks to develop a more comprehensive and context-sensitive understanding of the conditions that foster sustained SEL adoption.

## Figures and Tables

**Table 1:** Results of multiple regressions seeking to explain the use of SEL from exogenous variables.

	Behaviour	Intention	Attitude	Subjective Norm	Perceived Self-Efficacy	Perceived Controllability
**Experience**	−0.13 (0.01)	−0.10 (0.01)	−0.11 (0.01)	−0.13 (0.00)	−0.10 (0.01)	−0.09 (0.01)
**Males**	0.09 (0.14)	−0.05 (0.18)	−0.216 (0.16) *	−0.12 (0.11)	−0.02 (0.24)	−0.06 (0.12)
**More than 20% SSN ^1^**	0.16 (0.11) *	−0.09 (0.15)	0.17 (0.13) *	0.24 (0.09) **	−0.00 (0.20)	−0.09 (0.10)
**Grade level:**						
**Cycle 1**	0.20 (0.14) *	0.13 (0.17)	0.26 (0.15) *	0.27 (0.10) **	0.14 (0.23)	0.24 (0.12) *
**Cycle 3**	0.05 (0.15)	−0.01 (0.19)	0.07 (0.16)	0.09 (0.11)	0.03 (0.25)	0.08 (0.13)
**Cycle 4**	−0.05 (0.14)	0.13 (0.18)	−0.00 (0.16)	−0.06 (0.11)	−0.07 (0.24)	0.07 (0.12)
**Two cycles or more**	0.05 (0.30)	0.08 (0.38)	0.12 (0.34)	0.05 (0.23)	0.12 (0.51)	0.16 (0.26)
**School SES:**						
**Very low**	0.07 (0.17)	0.08 (0.22)	0.02 (0.19)	−0.04 (0.13)	−0.05 (0.29)	−0.12 (0.15)
**Low**	0.03 (0.14)	0.03 (0.19)	0.13 (0.17)	0.01 (0.11)	0.01 (0.25)	0.22 (0.13) *
**High**	0.05 (0.14)	−0.09 (0.18)	0.09 (0.16)	0.11 (0.11).	−0.10 (0.24)	0.09 (0.12)
**Very high**	0.04 (0.14)	−0.12 (0.18) *	0.01 (0.16)	0.04 (0.11)	−0.12 (0.24)	−0.06 (0.12)
**Attitude**	-	0.31 (0.22) *	-	-	-	-
**Subjective norm**	-	0.40 (0.30) **	-	-	-	-
**Perceived** **self-efficacy**	0.32 (0.06) ***	0.19 (0.08) **	-	-	-	-
**Perceived controllability**	−0.02 (0.10)	−0.07 (0.18)	-	-	-	-
**Intention**	0.37 (0.05) ***	-	-	-	-	-
**R^2^**	**51.8%**	**66.5%**	**16.4%**	**18.4%**	**6.2%**	**14.5%**

^1:^ SSN: students with special needs. * *p* < 0.05, ** *p* < 0.01, *** *p* < 0.001.

## Data Availability

The data that support the findings of this study are available from the corresponding author, M.T.-V., upon reasonable request.

## Abbreviations

The following abbreviations are used in this manuscript:

CASEL	Collaborative for Academic, Social, and Emotional Learning
CB-SEM	covariance-based structural equation modelling
CFA	confirmatory factor analysis
CFI	Comparative Fit Index
CR	composite reliability
ES	effect size
OECD	Organization for Economic Co-operation and Development
ONQS	National Observatory for School Quality
PATHS	Promoting Alternative Thinking Strategies
PLS	partial least squares
PLS-SEM	partial least squares structural equation modelling
RMSEA	root mean square error of approximation
RULER	recognizing, understanding, labelling, expressing, and regulating
SEL	social–emotional learning
SEM	structural equation modelling
SPSS	Statistical Package for the Social Sciences.
SRMR	standardised root mean square residual
SSN	students with special needs
STEM	science, technology, engineering, and mathematics
TPB	Theory of Planned Behaviour
TLI	Tucker–Lewis Index
VIF	variance inflation factor
WLSMV	weighted least squares mean and variance

## Appendix A

**Table A1:** Descriptive statistics of items measuring TPB variables in the context of SEL.

		Mean (sd)	1	2	3	4	5	6	7
	**ATTITUDES**								
att1	Developing students’ social-emotional and behavioral skills is useful.	-	0.6	0.0	0.6	7.2	27.7	56.6	-
att2	Developing students’ social and emotional and behavioral skills is an effective way to enhance academic learning.	-	0.0	0.6	1.8	19.3	33.1	36.7	-
att3	A positive behavioral climate in the classroom has a positive impact on students’ concentration and engagement.	-	0.0	0.0	0.0	7.1	27.3	65.6	-
att4	Developing students’ social and emotional and behavioral skills is generally a bad idea. ^R^	-	61.7	25.3	11	1.3	0.0	0.6	-
att5	Developing students’ social and emotional and behavioral skills is satisfying from a professional standpoint.	-	5.1	5.1	13.8	24.6	31.9	19.6	-
att6	Developing students’ social-emotional and behavioral skills is demotivating. ^R^	-	36.7	36.1	15.0	6.8	3.4	2.0	-
	**SUBJECTIVE NORMS**								
sn1	It is expected of me to develop students’ social-emotional and behavioral skills.	-	1.3	5.3	7.3	34.0	36.7	15.3	-
sn2	In my school cycle, activities aimed at the development of social and emotional and behavioral skills are perceived as relatively unimportant. ^R^	-	21.8	37.2	26.9	9.6	1.9	2.6	-
sn3	The individuals I esteem professionally foster students’ social and emotional and behavioral skills	-	0.7	2.1	5.5	32.4	38.6	20.7	-
	**PERCEIVED SELF-EFFICACY**								
sef1	Providing effective instruction for emotional learning (assisting students in recognizing and regulating their emotions and those of others).	4.05 (1.09)	-	-	-	-	-	-	-
sef2	Providing effective instruction for social learning (assisting students in establishing and maintaining healthy and positive relationships with others).	4.25 (0.92)	-	-	-	-	-	-	-
sef3	Providing effective instruction for behavioral learning (assisting students in recognizing and regulating positive behaviors and objectionable behaviors).	4.04 (1.05)	-	-	-	-	-	-	-
sef4	Providing effective instruction for responsible decision making (helping students make constructive choices in personal behavior and social interactions based on ethical standards, safety concerns, and social norms).	3.99 (1.10)	-	-	-	-	-	-	-
	**PERCEIVED CONTROLLABILITY**								
co1	It’s up to me to offer activities that develop students’ social-emotional and behavioral skills.	-	2.8	15.3	20.8	25.7	25.0	10.4	-
co2	I alone decide whether or not to propose activities that develop students’ social and emotional and behavioral skills.	-	3.4	7.5	16.4	29.5	30.8	12.3	-
co3	The current conditions in which I work do not allow for the development of students’ social-emotional and behavioral skills. ^R^	-	8.2	32.2	34.2	10.3	9.6	5.5	-
co4	The resources at my disposal are insufficient to propose activities to develop student’s social and emotional and behavioral skills. ^R^	-	5.5	21.2	26.7	24.0	13.7	8.9	-
	**INTENTION**								
int1	Implement social-emotional and behavioral skills teaching	4.35 (1.29)	-	-	-	-	-	-	-
int2	Teach students the knowledge, attitude, and skills needed to understand and manage the emotions of others.	4.49 (1.26)	-	-	-	-	-	-	-
tnt3	Implement teaching that develops students’ ability to make responsible personal, social, and academic decisions.	4.64 (1.19)	-	-	-	-	-	-	-
	**BEHAVIOUR**								
beh1	I teach my students to recognise and regulate their own emotions.	-	1.2	3.1	9.8	22.7	24.5	24.5	14.1
beh2	I ask my students to express their emotions in different ways (orally, through drawing, writing, games, puppets, etc.)	-	6.8	16.7	24.7	15.4	16.7	12.3	7.4
beh3	I organise social and emotional learning activities.	-	2.6	14.4	22.2	21.6	19.6	13.1	6.5
beh4	I teach my students to recognise and regulate their own behavior.	-	0.6	1.9	8.7	19.9	22.4	28.0	18.6
beh5	I teach my students to recognise emotions in others.	-	0.6	4.9	10.5	19.8	23.5	28.4	12.3
beh6	When a conflict arises in class or during recess, I ask students to share their emotions.	-	4.6	4.6	9.9	21.9	17.9	25.2	15.9
beh7	When a conflict arises in class or during recess, I ask students to first focus on the facts and then on their emotions.	-	0.6	2.5	12.5	14.4	23.1	22.5	24.4
beh8	I teach my students to work on emotions that arise in class (for example, after failing an assessment).	-	2.5	5.6	19.3	19.3	25.5	17.4	10.6
beh9	When a student is in difficulty, I try to find out if he or she has an emotional problem (boredom, anger, sadness, anxiety, etc.)	-	0.0	1.2	8.6	19.1	22.2	21.6	27.2
beh10	I use role-playing to boost my students’ self-confidence.	-	8.9	18.5	28.0	16.6	14.0	8.3	5.7

Attitude and subjective norm scales: 1 = not at all agree, 2 = disagree, 3 = somewhat disagree, 4 = somewhat agree, 5 = agree, 6 = strongly agree. Perceived controllability scale: 1 = not at all agree, 2 = disagree, 3 = somewhat disagree, 4 = somewhat agree, 5 = agree, 6 = strongly agree. Perceived self-efficacy scale: 0 = not at all competent, 6 = extremely competent. Intention scale: 0 = very little determined, 6 = very determined. Behaviour scale: 1 = never, 2 = rarely, 3 = sometimes, 4 = regularly, 5 = frequently, 6 = very frequently, 7 = systematically. ^R^ = Reverse-coded item.
